# FC-duplex IgG1 (HTLV-1/2): Qualifying the flow cytometry IgG1 duplex assay for differential diagnosis of HTLV-1 and HTLV-2 infections

**DOI:** 10.1371/journal.pntd.0013770

**Published:** 2025-12-08

**Authors:** Júlia Pereira Martins, Felipe Araujo Santos, Kelly Alves Bicalho, Jordana Grazziela Alves Coelho-dos-Reis, Vanessa Peruhype-Magalhães, Maísa Aparecida Ribeiro, Renata Glória Sá, Lucas Pedreira de Carvalho, Antonio Carlos Rosário Vallinoto, Laurence Rodrigues Amaral, Anna Barbara de Freitas Carneiro-Proietti, Andréa Teixeira-Carvalho, Allyson Guimarães Costa, Olindo Assis Martins-Filho, Márcio Sobreira Silva Araújo

**Affiliations:** 1 Grupo Integrado de Pesquisas em Biomarcadores de Diagnóstico e Monitoração, Instituto René Rachou – Fundação Oswaldo Cruz (FIOCRUZ) Minas, Belo Horizonte, Minas Gerais, Brazil; 2 Programa de Pós-Graduação em Imunologia Básica e Aplicada, Instituto de Ciências Biológicas, Universidade Federal do Amazonas (UFAM), Manaus, Amazonas, Brazil; 3 Diretoria de Ensino e Pesquisa, Fundação Hospitalar de Hematologia e Hemoterapia do Amazonas (HEMOAM), Manaus, Amazonas, Brazil; 4 Universidade Federal de Minas Gerais (UFMG), Belo Horizonte, Minas Gerais, Brazil; 5 Fundação Centro de Hematologia e Hemoterapia do Estado de Minas Gerais (HEMOMINAS), Belo Horizonte, Minas Gerais, Brazil; 6 Serviço de Imunologia, Universidade Federal da Bahia, Salvador, Bahia, Brazil; 7 Laboratório de Pesquisas Clínicas (LAPEC), Instituto Gonçalo Moniz, Fundação Oswaldo Cruz (FIOCRUZ) Salvador, Bahia, Brazil; 8 Laboratório de Virologia, Instituto de Ciências Biológicas, Universidade Federal do Pará, Belém, Pará, Brazil; 9 Laboratório de Bioinformática e Análises Moleculares, Universidade Federal de Uberlândia, Uberlândia, MG, Brazil; undefined, UNITED STATES OF AMERICA

## Abstract

Human T-lymphotropic virus (HTLV) infection has been implicated in a broad spectrum of clinical conditions, encompassing both neoplastic and inflammatory-degenerative disorders, associated with relevant immunological dysfunctions. Given the distinct epidemiological profiles and clinical manifestations associated with HTLV-1 and HTLV-2 infections, establishing an accurate differential diagnosis is of critical importance. The serological/molecular approaches currently available for differential diagnosis are expensive, time-consuming, and require multiple complementary assays, and still lead to inconclusive results. In this context, the development of a single-platform assay referred to as FC-Duplex IgG1 (HTLV-1/2) assay represented a valuable advance for the differential diagnosis of HTLV infections in clinical settings, while counseling HTLV-seropositive patients and/or conducting seroepidemiological surveys. The present study aimed at qualifying the FC-Duplex IgG1 (HTLV-1/2) kit prototype for differential serodiagnosis of HTLV-1 and HTLV-2 infections. A biorepository library, composed of serum samples from four HTLV reference centers (HTLV-1(+)/n = 225; HTLV-2(+)/n = 80 and HTLV1/2(-)/n = 55), was tested for anti-HTLV-1 and anti-HTLV-2 IgG1 reactivity using three classification criteria. Discriminant performance analysis supported the high accuracy of FC-Duplex IgG1 (HTLV-1/2) with elevated proportion of correct classification according to PCR & Western Blot reference standards (Criterion 3 = 89%; Criterion 2 = 90% and Criterion 1 = 99%). Decision tree accuracy and Leave One Out Cross-Validation re-enforced the robustness, confirming the minimal overfitting of our findings. Kappa agreement indices further confirmed the substantial to near perfect agreement of FC-Duplex IgG1 (HTLV-1/2) with reference standard methods (Criterion 3 = 0.72; Criterion 2 = 0.75 and Criterion 1 = 0.97). Altogether, these findings qualified the FC-Duplex IgG1 (HTLV-1/2) assay for differential diagnosis of HTLV-1/2 infections.

## Author summary

The HTLV infection can lead to very different clinical conditions based on which virus type is causing the infection and can impact a patient prognosis. An accurate and differential diagnosis being critical in this scenario, currently the methodologies used are often expensive, inconclusive and slow. To help solve this problem, we worked on developing a new methodology prototype called FC-Duplex IgG1 (HTLV-1/2) assay. This test is designed to be simpler and faster, while still giving accurate results. The results were promising: our test correctly identified HTLV-1 and HTLV-2 infections in most cases, matching the results of more complex and widely trusted methods. These findings suggest that our new test could be a useful tool for doctors and public health workers. It may help improve how we diagnose HTLV infections, provide better counseling to patients, and support efforts to track and manage the virus in communities.

## Introduction

Human T-lymphotropic virus (HTLV) is a retrovirus with a worldwide distribution [1]. The HTLV-1 and HTLV-2 types are the most prevalent and usually associated with human diseases. The World Health Organization estimates that 5–10 million people are infected with HTLV-1 and around 800,000 people are living with HTLV-2 [2]. While the HTLV-1 infection is widely distributed across all continents, the HTLV-2 infection occurs preferentially in endogenous populations, with dissemination to metropolitan areas recorded in the Americas, Europe, Africa, Asia, and Oceania [3]. In general, the HTLV infection can be transmitted through vertical, horizontal, and parenteral routes [4].

From a clinical perspective, most patients do not develop the hallmarks of clinical manifestations associated with HTLV-1 infection, such as HTLV-1-associated myelopathy/tropical spastic paraparesis (HAM/TSP) or adult T-cell leukemia/lymphoma (ATLL) [4]. However, several other clinical manifestations have been reported in HTLV-1-infected patients, including uveitis, severe eczema referred to as infective dermatitis, arthropathies, polymyositis, among others. While less pathogenic, the HTLV-2 infection has been associated with clinical conditions such as HAM/TSP-like disease, chronic lung inflammation, fibromyalgia, rheumatological manifestations, and increased cancer-related mortality [5–9]. Despite the clinical settings of several HTLV-associated diseases have been reported, the range of medical conditions associated with the HTLV infection is broader still, and several gaps remain to be addressed. The relevant changes in the host immune response elicited by the HTLV infection can impact on the susceptibility and clinical outcome of infectious-parasitic diseases in cases of co-infections. In this sense, the interactions between coexisting pathogens are complex and may affect the natural course of both infections [10].

Based on the above-mentioned epidemiological and clinical aspects, the accurate universal and differential diagnosis of HTLV infection is critical to subsidize the management of HTLV-1 and HTLV-2 infections and effectively control complications and improve the patient outcomes. In general, the diagnosis of HTLV-1/2 infection involves two steps: i) screening, ii) confirmatory and differential diagnosis. The screening usually employs highly sensitive serological tests with elevated negative predictive value, including: Chemiluminescence Immunoassay (CLIA) and enzyme-linked immunosorbent assays (ELISA), using viral lysates, recombinant proteins or synthetic peptides as target antigens. The confirmatory/differential diagnosis step generally employs tests of high specificity and positive predictive value, such as western blot (WB), line immunoassay (INNO-LIA), and polymerase chain reaction (PCR)-based molecular methods [11–13].

In general, the commercially available methods applicable for screening and confirmatory/differential diagnostic steps of HTLV infection present outstanding performance. However, the need for multi-step testing, the high costs, and the occurrence of inconclusive results represent a challenge to conclusively diagnose the HTLV infection in routine clinical laboratories. In this line, diagnostic approaches using new antigen targets on a single platform of competitive assays represent a technological advance for confirmatory and differential diagnosis of HTLV infection.

The development of a flow cytometry-based single platform for simultaneous measuring of anti-HTLV-1 and anti-HTLV-2 IgG1 reactivity applicable in the universal and differential serodiagnosis represented an innovation in the biotechnology segment with a high potential to compose a serological kit for differential diagnosis of HTLV-1/2 infection for reference laboratories and blood centers [14].

The present study intended to qualify a kit prototype of FC-Duplex IgG1 (HTLV-1/2) methodology for differential serodiagnosis of HTLV infections using three major steps: i) using a prototype in a clinical laboratory environment, ii) applying diagnosis criteria and iii) analyzing representative profiles & defining performance indices. Our findings provided evidence regarding the accuracy and agreement that qualified the FC-Duplex IgG1 (HTLV-1/2) assay for the differential diagnosis of HTLV-1/2 infections.

## Samples, materials and methods

### Ethics statement

The present study was approved by the Research Ethics Committee of the René Rachou Institute - FIOCRUZ/MG (C.A.A.E. N° 15047313.8.0000.5091) Ethical committee at HEMOAM (#739.563/2014) and Ethical Committee at HEMOMINAS (#4.639.423). The study fulfills the principles of the Helsinki declaration and the 466/2012 resolution of the Brazilian National Health Council for research involving human participants. All the participants read and signed the informed consent form (ICF) before enrollment.

### Biorepository library of serum samples and ethical approval

The present study comprises a multicentric investigation designed to qualify the laboratorial FC-Duplex IgG1 (HTLV-1/2) prototype for the differential diagnosis of HTLV-1 and HTLV-2 infections. For this purpose, a total of 360 serum samples archived in biorepository libraries from four Brazilian HTLV Reference Centers [Fundação Hospitalar de Hematologia e Hemoterapia do Amazonas (HEMOAM); Fundação Centro de Hematologia e Hemoterapia do Estado de Minas Gerais (HEMOMINAS); Laboratório de Pesquisas Clínicas from Instituto Gonçalo Muniz (LAPEC/FIOCRUZ-Bahia) and Universidade Federal do Pará (UFPA)] were tested using the laboratorial FC-Duplex IgG1 (HTLV-1/2) prototype. The biorepository library comprises specimens from patients with positive serology for HTLV-1/2 [HTLV-1/2 (+); n = 305] and blood donor candidates with negative serology for HTLV-1/2 infection and other infectious diseases tested during screening, considered reference controls [HTLV-1/2 (-); n = 55]. The serum samples included in the biorepository library were maintained at -80°C until processing.

The HTLV-1/2 (+) group compiled patients [HEMOAM (n = 22); HEMOMINAS (n = 145); LAPEC (n = 63) and UFPA (n = 75)], both sexes, aged from 2 to 86 years old, with positive serology for HTLV-1/2 infection performed by CLIA or ELISA, along with positive results by FC-Simplex IgG1 HTLV-1/2. The HTLV-1/2 (+) group was further classified as HTLV-1 (n = 225) or HTLV-2 (n = 80) based on reference standard methods: Western Blotting [WB (+)] or RT-qPCR [qPCR (+)].

The HTLV-1/2 (-) group comprised blood donor candidates [HEMOAM (n = 43) and HEMOMINAS (n = 12)], both sexes, aged from 18 to 50 years old, with negative serology for HTLV-1/2 infection performed by CLIA or ELISA along with negative results by FC-Simplex IgG1 HTLV-1/2.

This study was submitted and approved by the Research Ethics Committee from Instituto René Rachou - FIOCRUZ/MG (#4.255.156), HEMOAM (#5.348.608) and HEMOMINAS (#4.639.423). The study followed the guidelines and standards for research involving human beings, according to the resolution 466/2012 from the Brazilian National Health Council.

**Fig 1:**
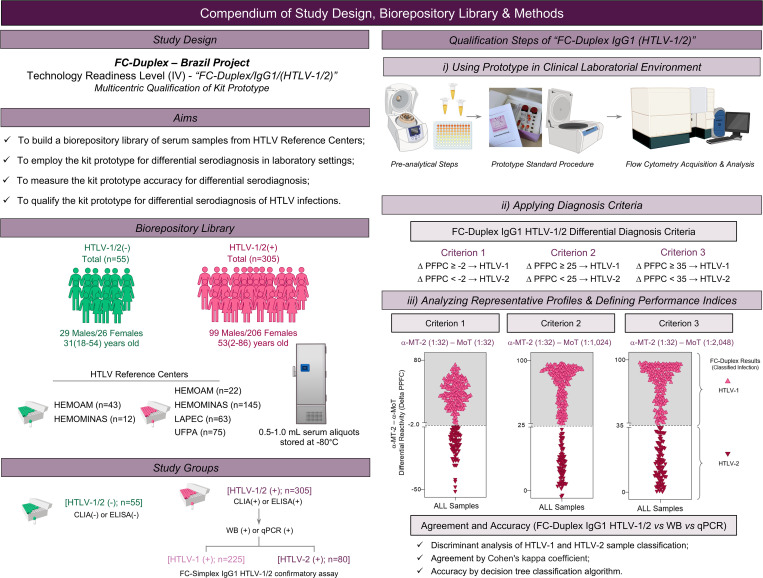
Compendium of study design, biorepository library & methods. The figure summarizes the five major milestones: “Study Design”; “Aims”; “Biorepository Library”; “Study Groups” and “Qualification Steps”. The qualification procedures comprised: i) using prototype in clinical laboratorial environment, ii) applying diagnosis criteria and iii) analyzing representative profiles & defining performance indices.

Fig 1 presents a compendium study design, aims, biorepository library, study groups, and qualification steps: i) using a prototype in a clinical laboratory environment, ii) applying diagnosis criteria, and iii) analyzing representative profiles & defining performance indices.

### FC-Simplex IgG1 (HTLV-1/2) confirmatory flow cytometry assay

**Fig 2:**
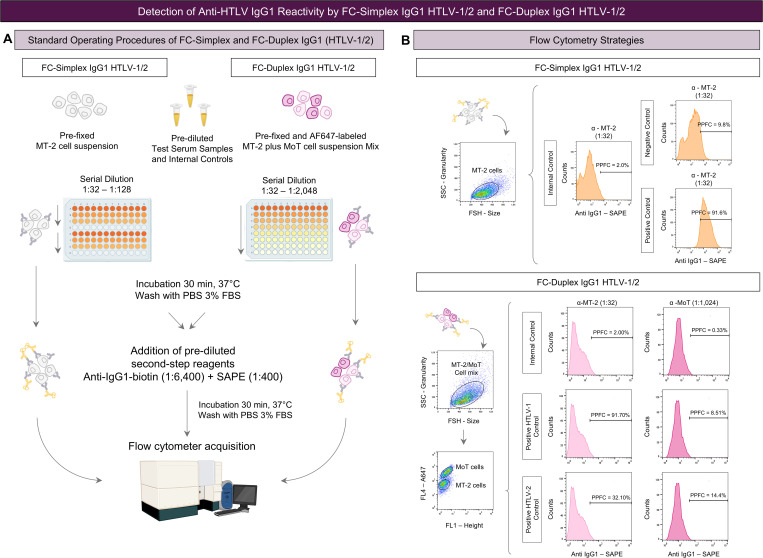
Detection of anti-HTLV IgG1 reactivity by FC-Simplex IgG1 HTLV-1/2 and FC-Duplex IgG1 HTLV-1/2. **(A)** Standard Operating Procedures of FC-Simplex and FC-Duplex IgG1 (HTLV-1/2). **(B)** Flow cytometry strategies to determine the anti-HTLV-1/2 IgG1 reactivity using the FC-Simplex IgG1 HTLV-1/2 and anti-HTLV-1/2 FC-Duplex IgG1 HTLV-1/2, illustrating the morphometric flow cytometry features of MT-2, MoT, and MT-2/MoT cell mix, the differential fluorescent staining of MT-2 and MoT cell lines along with the quantification of percentage of positive fluorescent cells (PPFC) based on the shift of FL2 fluorescence outside the positivity limit (PPFC < 2%) established for the internal control of secondary reagents.

The FC-Simplex IgG1 (HTLV-1/2) assay was performed as a qualitative methodology for confirmatory diagnosis of HTLV infection, as previously described [15]. The FC-Simplex IgG1 (HTLV-1/2) assay is a flow cytometry-based immunofluorescence platform to detect anti-HTLV-1/2 IgG1 antibodies, using the MT-2 cell line (commercially available at ATCC) as target antigen. Briefly, serum aliquots stored at -80°C were thawed at 37°C, heat-inactivated at 56°C for 30 min, and centrifuged at 17,000 x g at 4°C for 5 min to remove particles in suspension. Subsequently, twofold serial dilution of heat-inactivated serum samples (1:32–1:128) was prepared in PBS-3%. Fifty µL aliquots of pre-diluted serum samples were transferred to “U” bottom 96-well plates (Nunc, Roskilde, Denmark), followed by the addition 50 µL (1.0 × 10^5^ cells/well) of pre-fixed MT-2 cells (10 g/L of paraformaldehyde; 10.2 g/L of sodium cacodylate; 6.63 g/L of sodium chloride, pH 7.2). The plates were gently vortexed and incubated for 30 minutes at 37°C. After incubation, the cell suspension was washed twice (100 µL and 150 µL of PBS-3% FBS) by centrifugation at 400 × g for 10 minutes at 4°C. The supernatant was discarded, and the cell pellet resuspended with 50 µL of secondary reagents added to each well [biotin-labeled human anti-IgG1 (clone 8c/6–39, at 1:3,200 dilution in PBS) plus 10 µL of phycoerythrin-labeled streptavidin (SAPE, at 1:400 dilution in PBS)]. The plates were incubated for 30 min at 37°C protected from light. Following incubation, the cell suspension was washed twice using the abovementioned condition. The cell pellet was resuspended in 200 µL of PBS for acquisition in a FACSCalibur flow cytometer (BD Biosciences, San Jose, CA, USA). Internal controls of nonspecific binding of secondary reagents were carried out for each experimental batch. A total of 10,000 events were acquired for each sample. Data were stored using the CellQuest™ software package (BD Biosciences, San Jose, CA, USA). Data analysis was performed using the FlowJo Software (v10.1). The results were expressed as percentage of positive fluorescent cells (PPFC), based on the shift of FL2 fluorescence outside the positivity limit (PPFC < 2%) established for the internal control of secondary reagents. Fig 2 summarizes the major steps of FC-Simplex IgG1 (HTLV-1/2) assay.

### Qualification steps of FC-Duplex IgG1 (HTLV-1/2)

i) Using prototype in clinical laboratory environment

The FC-Duplex IgG1 (HTLV-1/2) prototype was employed as a serological method for differential diagnosis of HTLV-1 and HTLV-2 infections for samples presenting positive results in the FC-Simplex IgG1 (HTLV-1/2) assay, as previously described [14]. The FC-Duplex IgG1 (HTLV-1/2) assay is a flow cytometry-based single platform to simultaneously detect anti-HTLV-1 and anti-HTLV-2 IgG1 antibodies, employing MT-2 and MoT cell lines (commercially available at ATCC) as target antigens. Batches (1.0 × 10^8^ cells/mL in PBS) of pre-fixed MT-2 and MoT cell lines (10 g/L of paraformaldehyde; 10.2 g/L of sodium cacodylate; 6.63 g/L of sodium chloride, pH 7.2) were submitted to differential staining with Alexa Fluor 647 fluorescent dye (AF647) (0.002 μg/mL and 0.04 μg/mL; Thermo Fisher Scientific, MA, USA). Following quality control assessment by flow cytometry, a mixture of AF647-labeled MT-2 and MoT cell lines (1:1 Mix MT-2/MoT) was prepared to guarantee 50% proportion of each cell line and stored at 4°C up to seven days prior use. The standard operational procedure consisted of sequential steps as follows: serum aliquots stored at -80°C were thawed at 37°C, heat-inactivated at 56°C for 30 min and centrifuged at 17,000 x g at 4°C for 5 min to remove particles in suspension. Twofold serial dilution of heat-inactivated serum samples (1:32–1:2,048) was prepared in PBS-3%. Fifty µL aliquots of pre-diluted serum samples were transferred to “U” bottom 96-well plates (Nunc, Roskilde, Denmark), followed by the addition 50 µL (1.0 × 10^5^ cells/well) of Mix MT-2/MoT. The plates were gently vortexed and incubated for 30 minutes at 37°C. After incubation, the cell mix suspension was washed twice (100 µL and 150 µL of PBS-3% FBS) by centrifugation at 400 × g for 10 minutes at 4°C. The supernatant was discarded, and the cell mix pellet resuspended with 50 µL of secondary reagents added to each well [biotin-labeled human anti-IgG1 (clone 8c/6–39, at 1:3,200 dilution in PBS) plus 10 µL of phycoerythrin-labeled streptavidin (SAPE, at 1:400 dilution in PBS)]. The plates were incubated for 30 min at 37°C, protected from light. Thereafter, the cell mix suspension was washed twice as aforementioned. The cell mix was resuspended in 200 µL of PBS for acquisition in a FACSCalibur flow cytometer (BD Biosciences, San Jose, CA, USA). Internal controls of nonspecific binding of secondary reagents were carried out for each experimental batch. A total of 10,000 events were acquired for each sample. Data were stored using the CellQuest™ software package (BD Biosciences, San Jose, CA, USA). Data analysis was performed using the FlowJo Software (v10.1). The results were expressed as percentage of positive fluorescent cells (PPFC), based on the shift of FL2 fluorescence outside the positivity limit (PPFC < 2%) established for the internal control of secondary reagents. Fig 2 summarizes the major steps of FC-Duplex IgG1 (HTLV-1/2) assay.

ii) Applying differential diagnosis criteria using FC-Duplex IgG1 (HTLV-1/2) assay

The differential diagnosis of HTLV-1 and HTLV-2 infections was accomplished using the FC-Duplex IgG1 (HTLV-1/2) assay, employing synchronous and asynchronous pairwise serum dilutions to calculate the difference between the anti-MT-2 and anti-MoT IgG1 reactivity (Delta PPFC = %MT-2 – %MoT) along the titration curves (1:32–1:2048), as previously described [14]. Three differential diagnosis criteria were applied as follows: Criterion 1 = synchronous pairing [(α-MT-2 (1:32) – α-MoT (1:32)]; Criterion 2 = asynchronous pairing [α-MT-2 (1:32) – α-MoT (1:1,024)] and Criterion 3 = asynchronous pairing [α-MT-2 (1:32) – α-MoT (1:2,048)] strategies. The following cut-offs of delta PPFC between anti-MT-2 and anti-MoT cell lines (ΔPPFC) were used: Criterion 1 (Δ PPFC ≥ -2 → HTLV-1 and Δ PPFC < -2 → HTLV-2); Criterion 2 (Δ PPFC ≥ 25 → HTLV-1 and Δ PPFC < 25 → HTLV-2), and Criterion 3 (Δ PPFC ≥ 35 → HTLV-1 and Δ PPFC < 35 → HTLV-2).

iii) Defining performance indices for FC-Duplex IgG1 (HTLV-1/2) assa*y*

The performance of FC-Duplex IgG1 (HTLV-1/2) assay was assessed by discriminant analysis of HTLV-1 and HTLV-2 sample classification, agreement assessment using Cohen’s kappa coefficient and accuracy analysis employing decision tree classification algorithm.

Discriminant analysis of FC-Duplex IgG1 (HTLV-1/2) was accomplished, comparing the correct HTLV-1 and HTLV-2 classification according to PCR & Western Blot reference standards. Additionally, the Cohen’s Kappa coefficient (*Kappa* index = *k*) was calculated with 95% confidence interval, and interpreted according to [16] as follows: no agreement (κ < 0); slight (κ > 0.00 ≤ 0.20); fair (κ ≥ 0.21 ≤ 0.40); moderate (κ ≥ 0.41 ≤ 0.60); substantial (κ ≥ 0.61 ≤ 0.80); almost perfect agreement (κ ≥ 0.81 ≤ 1.00). Prisma GraphPad software version v.8.1 (GraphPad Prism Software, San Diego, CA, USA) was used for statistical analysis and graphical arts.

Decision tree algorithms were constructed to determine the full training accuracy of FC-Duplex IgG1 (HTLV-1/2) to classify HTLV-1 and HTLV- 2 individual samples for differential diagnosis purposes, using Criterion 1, Criterion 2 and Criterion 3. “Leave One Out Cross-Validation” (LOOCV) was applied to estimate the performance of FC-Duplex IgG1 (HTLV-1/2) creating multiple training sets from the original data. The WEKA software, version 3.6.11 (University of Waikato, New Zealand), was used for decision tree construction and LOOCV analysis.

## Results

### FC-Simplex IgG1 (HTLV-1/2) confirmatory flow cytometry assay for serum samples archived in the biorepository library

**Fig 3:**
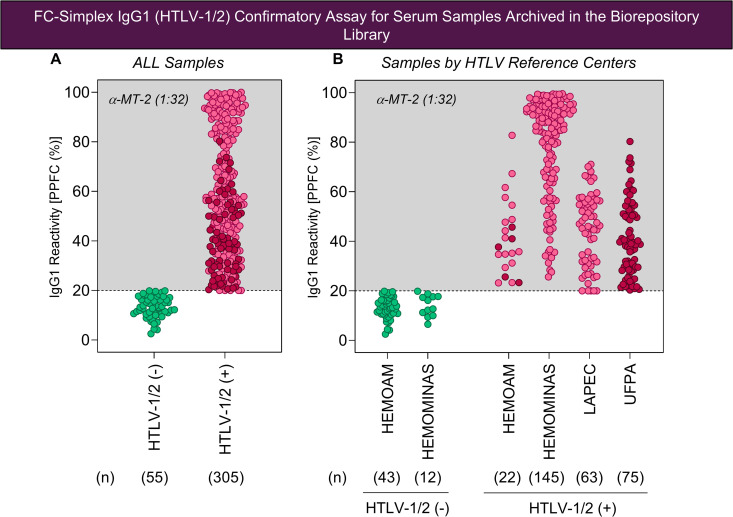
FC-Simplex IgG1 (HTLV-1/2) confirmatory assay for serum samples archived in the biorepository library. Serum samples (n = 360) from biorepository library of four Brazilian HTLV Reference Centers (HEMOAM, HEMOMINAS, LAPEC and UFPA) were processed to confirm the HTLV reactivity. The FC-Simplex IgG1 (HTLV-1/2) results are presented as scattering distribution of individual values expressed as Percentage of Positive Fluorescent Cells [PPFC (%)] in serum dilution (1:32) for HTLV-1/2 (-) controls (, n = 55) and HTLV-1/2 (+) samples (, n = 305), comprising: (A) all samples and (B) samples by HTLV Reference Centers. The dashed line represents the PPFC value of 20% used to classify negative (PPFC ≤ 20%) and positive samples (PPFC > 20%).

Serum samples from the biorepository library were screened using the FC-Simplex IgG1 (HTLV-1/2) assay to accomplish the confirmatory diagnosis of HTLV infection. The results are presented in Fig 3.

The FC-Simplex IgG1 (HTLV-1/2) assay is employed for universal diagnosis of HTLV infection based on the anti-HTLV-1/2 IgG1 antibodies reactivity, using the MT-2 cell line as target antigen and the cut-off limit of PPFC = 20% to classify serum samples as negative (PPFC < 20%) or positive (PPFC ≥ 20%) for HTLV infection. The results demonstrated that 55 out of 360 samples archived in the biorepository library (15.3%) were confirmed as negative for HTLV infection, presenting IgG1 reactivity below 20%. On the other hand, 305 out of 360 archived samples (84.7%) were confirmed as positive for HTLV infection, exhibiting IgG1 reactivity above or equal to 20% PPFC (Fig 3A). In depth analysis of IgG1 reactivity by FC-Simplex IgG1 (HTLV-1/2) assay demonstrated that PPFC values ranged from 2.5-19% for negative samples and from 20-100% for positive samples.

Data analysis by HTLV Reference Centers demonstrated low IgG1 reactivity for serum samples from HEMOAM, LAPEC and UPFA, with PPFC values ranging from 23-83%, 20–71% and 20–80% and median values of 39% (IQR = 32–49%), 46% (IQR = 32–56%) and 39% (IQR = 29–52%), respectively. Samples from HEMOMINAS high IgG1 reactivity, with PPFC values ranging from 26-100% and median values of 87% (IQR = 66–94%) (Fig 3B). These differences in IgG1 reactivity for serum samples tested by FC-Simplex IgG1 (HTLV-1/2) assay may reflect the distinct proportion of samples from HTLV-1 and HTLV-2 archived in each biorepository library or may reflect the clinical forms of HTLV infected patients.

### Differential diagnosis of serum samples FC-Duplex IgG1 (HTLV-1/2) prototype in clinical laboratorial environment

**Fig 4:**
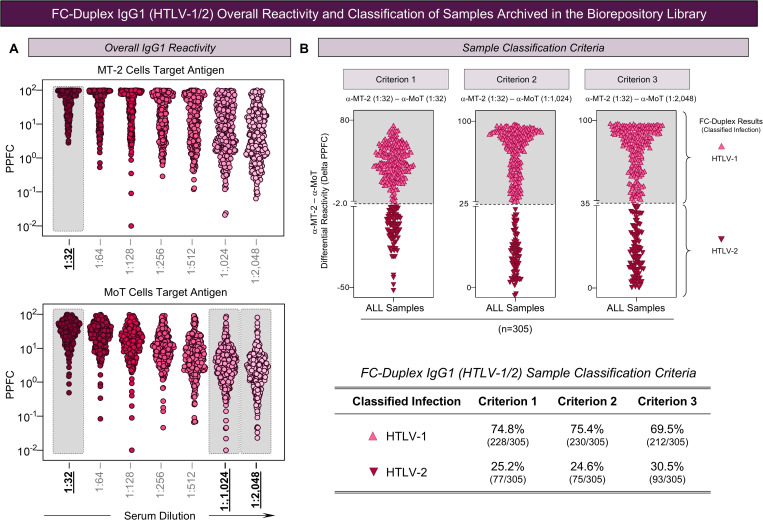
FC-Duplex IgG1 (HTLV-1/2) overall reactivity and classification of samples archived in the biorepository library. **(A)** Overall IgG1 reactivity with MT-2 (top panel) and MoT cell lines (bottom panel) along the titration curve (1:32 to 1:2,048). The results are presented as scattering distribution of individual values expressed as Percentage of Positive Fluorescent Cells [PPFC (%)] at each serum dilution, for samples presenting positive results in FC-Simplex IgG1 (HTLV-1/2) confirmatory assay. The gray rectangles underscore the serum dilutions employed for synchronous and asynchronous pairwise analysis to calculate the difference between the anti-MT-2 and anti-MoT IgG1 reactivity (Delta PPFC = %MT-2 – %MoT). **(B)** Classification of samples by FC-Duplex IgG1 (HTLV-1/2) assay to accomplish the differential diagnosis of HTLV-1 and HTLV-2 infections. Three differential diagnosis criteria were applied as follows: Criterion 1 = synchronous pairing [(α-MT-2 (1:32) – α-MoT (1:32)]; Criterion 2 = asynchronous pairing [α-MT-2 (1:32) – α-MoT (1:1,024)] and Criterion 3 = asynchronous pairing [α-MT-2 (1:32) – α-MoT (1:2,048)] strategies. The following cut-offs of delta PPFC between anti-MT-2 and anti-MoT cell lines (ΔPPFC) were used: Criterion 1 (Δ PPFC ≥ -2 → HTLV-1 and Δ PPFC < -2 → HTLV-2); Criterion 2 (Δ PPFC ≥ 25 → HTLV-1 and Δ PPFC < 25 → HTLV-2) and Criterion 3 (Δ PPFC ≥ 35 → HTLV-1 and Δ PPFC < 35 → HTLV-2). The inserted table provides the final sample classification using each criterion.

The differential diagnosis of HTLV infection was accomplished using the FC-Duplex IgG1 (HTLV-1/2) prototype. For this purpose, serum samples with positive anti-HTLV-1/2 IgG1 antibodies reactivity in the FC-Simplex IgG1 (HTLV-1/2) assay were tested using the FC-Duplex IgG1 (HTLV-1/2) prototype in clinical laboratorial environment. The results are presented in Fig 4.

Initially, the overall IgG1 reactivity to MT-2 and MoT cells target antigens was determined along the titration curve, employing distinct serum dilutions (1:32–1:2,048). The IgG1 reactivity at specific serum dilutions (1:32 for MT-2 and 1:32, 1:1,024, and 1:2,048 for MoT target antigens) was selected for synchronous and asynchronous pairwise analysis used in the differential diagnosis criteria as previously proposed [14] (Fig 4A).

According to three differential diagnosis criteria used in the FC-Duplex IgG1 (HTLV-1/2), synchronous [(α-MT-2 (1:32) – α-MoT (1:32)] and asynchronous pairwise analysis [(α-MT-2 (1:32) – α-MoT (1:1,024) and α-MT-2 (1:32) – α-MoT (1:2,048)] were employed to calculate the Delta IgG1 reactivities (Delta PPFC = %MT-2 – %MoT) to classify the HTLV infection using pre-established cut-off thresholds (Delta PPFC = -2%, 25% and 35% respectively)(Fig 4B). The results demonstrated that, according to the Criterion 1, 228 out of 305 samples (74.8%) were classified as HTLV-1 infection, with median Delta PPFC values of 36.9 (Min = -1.8; Max = 74.7; IQR = 26.7 to 50.3). On the other hand, 77 out of 305 samples (25.2%) were diagnosed as HTLV-2 infection, with median Delta PPFC values of -13.2 (Min = -51.8; Max = -2.2; IQR = -20.6 to -7.9) (Fig 4B).

Based on the Criterion 2, 75.4% of the tested samples (230/305) were classified as HTLV-1 infection, with median Delta PPFC values of 79.4 (Min = 25.2; Max = 97.1; IQR = 55.0 to 89.2). A total of 24.6% samples (75/305) were diagnosed as HTLV-2 infection; with median Delta PPFC values of 9.8 (Min = -2.4; Max = 24.0; IQR = 5.3 to 14.1) (Fig 4B).

The use of Criterion 3 classified 69.5% of the tested samples (212/305) as HTLV-1 infection, and 30.5% of the tested samples (93/305) as HTLV-2 infection. The median Delta PPFC values of 83.5 (Min = 35.1; Max = 97.5; IQR = 63.4 to 91.9) for samples classified as HTLV-1 infection and median Delta PPFC values of 13.4 (Min = 0.1; Max = 34.7; IQR = 7.9 to 22.9) for samples classified as HTLV-2 infection (Fig 4B).

Overall, regardless of the diagnosis criteria employed (Criterion 1, Criterion 2, and Criterion 3), no significant differences were observed in the proportion of samples classified as HTLV-1 (74.8%, 75.4% and 69.5%, respectively) or HTLV-2 infections (25.2%, 24.6% and 30.5%, respectively) (Fig 4, inserted Table).

### Performance indices of FC-Duplex IgG1 (HTLV-1/2) classification according to PCR & Western Blot results

**Fig 5:**
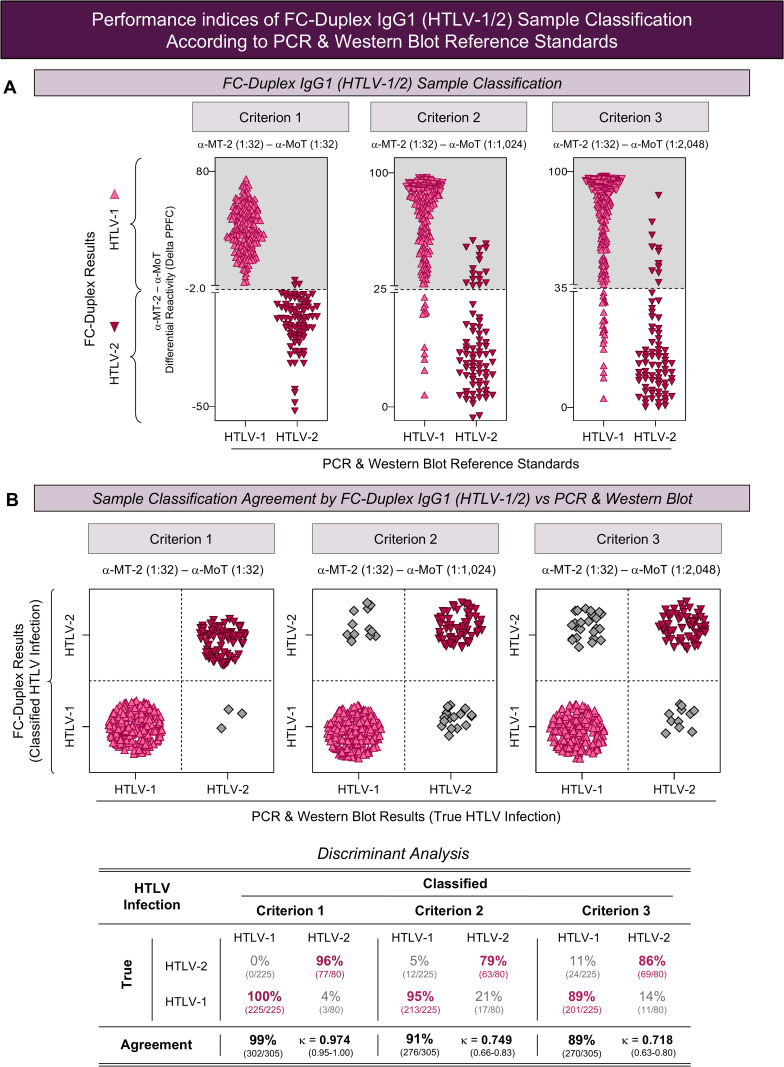
Performance indices of FC-Duplex IgG1 (HTLV-1/2) sample classification according to PCR & Western Blot reference standards. **(A)** FC-Duplex IgG1 (HTLV-1/2) sample classification according to PCR & Western Blot reference standards, applying differential diagnosis criteria as follows: Criterion 1 = synchronous pairing [(α-MT-2 (1:32) – α-MoT (1:32)]; Criterion 2 = asynchronous pairing [α-MT-2 (1:32) – α-MoT (1:1,024)} and Criterion 3 = asynchronous pairing [α-MT-2 (1:32) – α-MoT (1:2,048)]. The gray backgrounds underscore the samples classified as HTLV-1 (▲), considering: Criterion 1 (Δ PPFC ≥ -2); Criterion 2 (Δ PPFC ≥ 25) and Criterion 3 (Δ PPFC ≥ 35). The white backgrounds underscore the samples classified as HTLV-2 (▼), considering: Criterion 1 (Δ PPFC < -2), Criterion 2 (Δ PPFC < 25) and Criterion 3 (Δ PPFC < 35) cut-offs in FC-Duplex IgG1 (HTLV-1/2). **(B)** Sample classification agreement between FC-Duplex IgG1 (HTLV-1/2), PCR & Western Blot reference standards using the differential diagnosis criteria. Data are presented in bi-dimensional plots comparing HTLV-1 and HTLV-2 classification by PCR & Western Blot reference standards (True HTLV Infection) with FC-Duplex IgG1 (HTLV-1/2) (Classified HTLV Infection). True-classification of samples as HTLV-1 infection (▲) and HTLV-2 (▼) are clustered apart from misclassified samples (◆). Detailed discriminant analysis and Cohen’s Kappa coefficient (*Kappa* index = κ) are provided in the inserted table.

Aiming at evaluating the performance of FC-Duplex IgG1 (HTLV-1/2) prototype to classify serum samples in clinical laboratorial environment, the differential diagnosis accomplished by Criterion 1, 2, and 3 were compared with the results obtained by PCR and Western Blot reference standards methods. The results are shown in Fig 5.

Data demonstrated that based on the Criterion 1 [α-MT-2 (1:32) – α-MoT (1:32)], all samples previously defined as HTLV-1 infection by reference standard methods (n = 225) were correctly classified as HTLV-1 by FC-Duplex IgG1 (HTLV-1/2). Conversely, 77/80 samples (96%) defined as HTLV-2 by reference standard methods were classified as HTLV-2 by FC-Duplex IgG1 (HTLV-1/2). A total of 3 misclassifications were reported for the Criterion 1 (Fig 5A and 5B).

According to the Criterion 2 [α-MT-2 (1:32) – α-MoT (1:1,024)], 213/225 samples (95%) defined as HTLV-1 by reference standard methods were confirmed as HTLV-1 by FC-Duplex IgG1 (HTLV-1/2). A total of 63/80 (79%) of HTLV-2 samples were confirmed positive by FC-Duplex IgG1 (HTLV-1/2). A total of 29 misclassifications were reported for the Criterion 2, 12 misidentified as HTLV-1 and 17 as HTLV-2 (Fig 5A and 5B).

Applying the Criterion 3 [α-MT-2 (1:32) – α-MoT (1:2,048)], the results demonstrated that 201 out of 225 (89%) HTLV-1 samples were classified correctly by FC-Duplex IgG1 (HTLV-1/2), while 69/80 (86%) were classified as HTLV-2 infection. A total of 35 misidentifications were reported for the Criterion 3, 24 misclassified as HTLV-1 and 11 as HTLV-2 (Fig 5A and 5B).

Discriminant analysis demonstrated that the Criterion 1 reached an overall agreement of 99% (302/305) with Cohen’s Kappa coefficient of 0.974 (0.95-1.00). On the other hand, the Criterion 2 yielded an overall agreement of 91% [276/305; κ = 0.749 (0.66-0.83)], whereas the Criterion 3 exhibited an agreement of 89% (270/305) with κ = 0.718 (0.63-0.80) (Fig 5, inserted Table).

Overall, the FC-Duplex IgG1 (HTLV-1/2) prototype was qualified with outstanding classification agreements supported by Cohen’s Kappa agreement coefficients considered almost perfect (κ ≥ 0.81 ≤ 1.00) for the Criterion 1 and as substantial (κ ≥ 0.61 ≤ 0.80) for Criterion 2 and 3 (Fig 5, inserted Table).

### FC-Duplex IgG1 (HTLV-1/2) accuracy for differential diagnosis of HTLV infections

**Fig 6:**
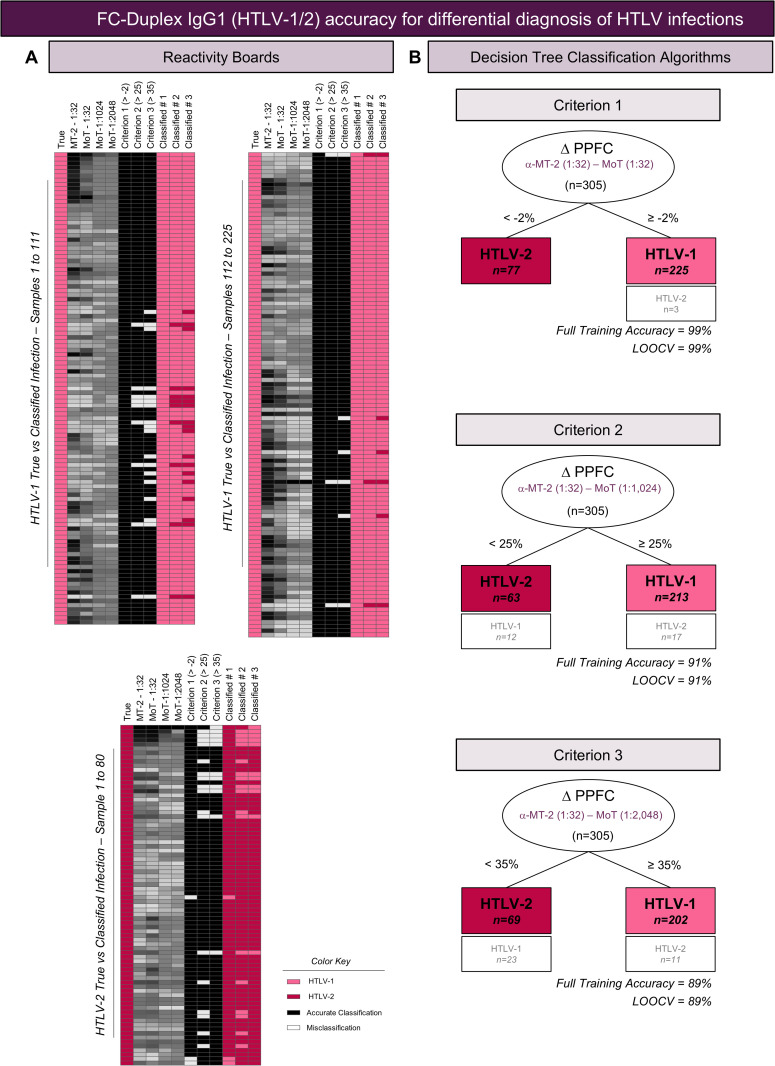
FC-Duplex IgG1 (HTLV-1/2) accuracy for differential diagnosis of HTLV infections. **(A)** Reactivity boards of “True” HTLV Infection and “Classified” results obtained for HTLV-1 (top panel) and HTLV-2 samples (bottom panel). “True” HTLV Infection compromises classification performed by the PCR & Western Blot reference standards. “Classified” results consisted of the classification achieved by FC-Duplex IgG1 (HTLV-1/2) assay, applying three differential diagnosis criteria. **(B)** Decision tree algorithm was built using pre-selected set of attributes applied for differential diagnosis according to the following criteria: Criterion 1 – Delta [α-MT-2 (1:32) – α-MoT (1:32)] & cut-off limit of PPFC = -2; Criterion 2 – Delta [α-MT-2 (1:32) – α-MoT (1:1,024)] & cut-off limit of PPFC = 25 and Criterion 3 – Delta [α-MT-2 (1:32) – α-MoT (1:2,048)] & cut-off limit of PPFC = 35. The classification root yield decision leaves for classification of HTLV infections. The number of correct results and misclassifications of samples ranked on each leaf were defined by machine learning models and considered to calculate the full training accuracy. The “leave-one-out-cross-validation” (LOOCV) values were created for multiple training sets from the original data and provided in the figure.

To further validate the FC-Duplex IgG1 (HTLV-1/2) prototype for use in clinical laboratorial environment, the results were compiled into reactivity boards of “True” and “Classified” HTLV-1 and HTLV-2 infections and decision tree algorithms proposed for sample classification. The results are presented in Fig 6.

The results assembled into reactivity boards provided a panoramic snapshot of data obtained for individual samples defined as “True” infections by reference standard methods (PCR & Western Blot) and “Classified” infections by FC-Duplex IgG1 (HTLV-1/2) prototype (Fig 6A). The reactivity boards illustrated that, as for HTLV-1 infection, higher correspondence was observed between “True” vs “Classified” infections defined by the Criterion 1, while lower similarity was observed for the Criterion 3. Likewise, the Criterion 1 exhibited higher similarity between “Classified” “True” HTLV-2 infection, whereas minor correspondence was observed for Criterion 3 and even lower for Criterion 2 (Fig 6A).

Decision tree analysis, employing the results obtained for individual samples and the three differential diagnosis criteria (Criterion 1, 2 and 3) and the respective cut-off thresholds (Delta PPFC = -2, 25 and 35) demonstrated high full training accuracy for FC-Duplex IgG1 (HTLV-1/2) prototype in distinguishing between well characterized HTLV-1/2 samples (99%, 91% and 89%, respectively). “Leave One Out Cross-Validation” (LOOCV), applied to create multiple training sets from the original data, confirmed the elevated accuracy of FC-Duplex IgG1 (HTLV-1/2) prototype for differential diagnosis of HTLV infections (Fig 6B).

## Discussion

Despite the HTLV infection being associated with serious clinical manifestations in humans, there is still a gap between the diagnosis and prevention in many countries, highlighting the need for improvements in the current established methods and protocols. In Brazil, serological screening became mandatory in blood banks in 1993, and the Ministry of Health recommends that the diagnosis of HTLV infection be made through two stages: screening, in which serological tests are used, and confirmatory tests, in addition to serological and molecular tests such as Western Blot and PCR are also used [11,12,17]. In Brazil, as in other countries, only blood donors undergo mandatory screening tests, which makes it difficult to identify, treat, and contain transmission. In addition, there is still no gold standard test for HTLV, and all confirmatory tests have limitations, which makes it difficult to guide reactive individuals [18].

Due to the failure of current diagnostic methods in performing the differential diagnosis of HTLV 1/2 infection, it is essential to seek alternative methods of diagnosis. Previous studies by our group have revealed that flow cytometry can be an important methodology for the detection of immunoglobulins specific for HTLV [14,15,19,20] and also for other infectious diseases of importance to public health [21–26].

Complementing previous studies carried out by our group [14], in this work we propose to qualify the performance of the Laboratorial FC-Duplex IgG1 (HTLV-1/2) prototype for the differential diagnosis of HTLV-1 and HTLV-2 infections. A multicentric biorepository library of serum samples from individuals with laboratory diagnosis of HTLV-1 and HTLV-2 infections, as well as from uninfected individuals (control group) received from four HTLV reference centers from Brazil, were employed to determine the agreement of the FC-Duplex IgG1 (HTLV-1/2) with the reference standards Western Blot and PCR.

Our results presented excellent performance for the differential diagnosis of HTLV-1 and HTLV-2. The evaluation was realized with 305 serum samples from individuals with a previous confirmed diagnosis of HTLV-1 (n = 225) and HTLV-2 (n = 80), and HTLV-1/2 negative controls (n = 55) from four HTLV reference centers from distinct Brazilian regions. An initial confirmatory screening employing the FC-Simplex IgG1 (HTLV-1/2) assay was able to differentiate the HTLV-1/2 (-) from HTLV-1/2 (+) group, the universal diagnosis of HTLV infection based on the anti-HTLV-1/2 IgG1 antibodies reactivity, using the MT-2 cell line as target antigen and the cut-off limit of PPFC = 20% to classify serum samples as negative (PPFC < 20%) or positive (PPFC ≥ 20%) for HTLV infection. The IgG1 reactivity profile observed in the data analysis by individual HTLV reference centers reflects different profiles between the HTLV-1 and HTLV-2 positive samples of each biorepository, with HTLV-1 positive samples generally presenting higher PPFC. The differences in IgG1 reactivity in the FC-Simplex IgG1 (HTLV-1/2) assay can reflect the immunological profile and proviral load in the HTLV infected patients. A study from our colleagues [15] demonstrated that serum samples (1:1,024 vs. 1:4096) from asymptomatic and possible/putative HAM/TSP (pHAM) patients with double positive reactivity with MT-2 cells had higher IFN-γ and proviral load, the asymptomatic group with double positive profile also presented lower IL-10, while the double positive pHAM group displayed higher TNF. The proviral load and cytokine profile were also analyzed for HTLV-1 HAM/TSP, one of the most serious clinical manifestations from the HTLV-1 infection, and although this group displayed lower TNF and IL-10 they had higher proviral load, with the authors concluding that MT-2 IgG1 reactivity profile correlates with proviral load, with significant positive association. These findings could explain the different IgG1 reactivity noticed in the HTLV-1 and HTLV-2 confirmed samples, but at this time, we do not have access to the clinical profile of the HTLV positive patients tested in this study.

The positive samples in the confirmatory assay were then analyzed using the three criteria for the FC-Duplex IgG1 (HTLV-1/2), further classifying correctly (99%; 90% and 89%) HTLV-1 and HTLV-2 positive samples when compared to previous results of the reference standards Western Blot and PCR. The results obtained in the tests carried out reinforce that the laboratorial FC-Duplex IgG1 (HTLV-1/2) prototype proved to be promising for use in diagnostic services for blood centers and reference centers in the differential diagnosis of HTLV, while the FC-Simplex IgG1 (HTLV-1/2) can be applied as screening assay for the universal diagnosis of the infections.

One significant advantage of the FC-Duplex IgG1 (HTLV-1/2) assay stands in the design, which incorporates a highly sensitive detection system (Biotin/Streptavidin/Phycoerythrin) that enables efficient IgG1 binding detection via flow cytometry, even at high serum dilutions. Additionally, this method has strong potential for automation, utilizes microvolumes, and can be easily implemented in other laboratories, making it highly adaptable for broader diagnostic applications.

However, this study also has some limitations. The unequal distribution of negative and positive samples, as well as the imbalance in HTLV-1 and HTLV-2 sample representation, may introduce bias when evaluating diagnostic performance based on the viral type. Furthermore, the study did not include samples from individuals with other known viral co-infections, such as hepatitis A virus (HAV), HBV, and HCV, which could potentially introduce cross-reactivity and impact diagnostic accuracy.

We observed that the FC-Duplex IgG1 (HTLV-1/2) assay correctly classified 89–99% of the HTLV-1 or HTLV-2 tested samples using the three pre-established criteria’s with Criterion 1 being the most accurate of the three, this contrasts our previous study by [14] which indicated as the most reliable pairwise condition to segregate HTLV-1 from HTLV-2 using the Criteria 2 Although those results indicate an sensitive and specific results, false-positive and false-negative results still a major challenge in immunological assays. False results can heavily impact the quality of life, mental health of the patients, and could also delay health assistance for the positive cases, further improving the methodology to lower the frequency of false results can strengthen the trustworthiness of the FC-Duplex IgG1 (HTLV-1/2) assay. Overall, the FC-Duplex IgG1 (HTLV-1/2) assay shows considerable promise as a tool for accurate differential diagnosis. With further validation using a broader range of sample conditions, its clinical utility can be further solidified, enhancing its potential for routine diagnostic use.

The primary strength of this study lies in its multicenter design, incorporating samples from four HTLV reference centers constructing a biorepository library with well-characterized HTLV-1/2 samples confirmed by reference standards Western blot and PCR. The differential diagnosis achieved using the FC-Duplex IgG1 (HTLV-1/2) assay was validated through concordant results with samples previously confirmed by standard diagnostic methods with a substantial and almost perfect agreement (κ = 0.974; 0.749; 0.718) in each of the three diagnostic criteria, demonstrating reliability of the proposed prototype.

In conclusion, this study, a new serological method for the diagnosis of HTLV infection, the FC-Duplex IgG1 (HTLV-1/2), was evaluated and the results showed it to be an efficient method for diagnosis and to differentiate the infection into HTLV-1 and HTLV-2. The method demonstrated high performance and applicability for the universal and differential diagnosis of human HTLV-1/2 infection, filling a gap in the differential diagnosis of human HTLV infection.
