# Supplementary material for: FC-duplex IgG1 (HTLV-1/2): Qualifying the flow cytometry IgG1 duplex assay for differential diagnosis of HTLV-1 and HTLV-2 infections
Source: PLoS Negl Trop Dis. 2025 Dec 8;19(12):e0013770. doi: 10.1371/journal.pntd.0013770 (PMC12714213; doi:10.1371/journal.pntd.0013770)
Supplement: Peer review history

# Editor report: Decision Letter 0

**DOI:** 10.1371/journal.pntd.0013770.r001

**Authors:** Angelica Arcanjo

18 Nov 2025

Dear Dr Araujo,

We are pleased to inform you that your manuscript 'FC-Duplex IgG1 (HTLV-1/2): Qualifying the Flow Cytometry IgG1 Duplex Assay for differential diagnosis of HTLV-1 and HTLV-2 Infections' has been provisionally accepted for publication in PLOS Neglected Tropical Diseases.

Before your manuscript can be formally accepted you will need to complete some formatting changes, which you will receive in a follow up email. A member of our team will be in touch with a set of requests.

Please note that your manuscript will not be scheduled for publication until you have made the required changes, so a swift response is appreciated.

IMPORTANT: The editorial review process is now complete. PLOS will only permit corrections to spelling, formatting or significant scientific errors from this point onwards. Requests for major changes, or any which affect the scientific understanding of your work, will cause delays to the publication date of your manuscript.

Should you, your institution's press office or the journal office choose to press release your paper, you will automatically be opted out of early publication. We ask that you notify us now if you or your institution is planning to press release the article. All press must be co-ordinated with PLOS.

Thank you again for supporting Open Access publishing; we are looking forward to publishing your work in PLOS Neglected Tropical Diseases.

Best regards,

Angelica Arcanjo

Guest Editor

PLOS Neglected Tropical Diseases

Sujatha Sunil

Section Editor

PLOS Neglected Tropical Diseases

Shaden Kamhawi

co-Editor-in-Chief

PLOS Neglected Tropical Diseases

orcid.org/0000-0003-4304-636XX

Paul Brindley

co-Editor-in-Chief

PLOS Neglected Tropical Diseases

orcid.org/0000-0003-1765-0002

***********************************************************

The work in question is quite clear in presenting its methodology and results; the number of samples used is relevant and representative since it includes samples from different states of the country and successfully achieves its objective by proposing a new technique for HTLV diagnosis. However, I am still not convinced whether this methodology is practical enough to be used in diagnostic centers.

p.p1 {margin: 0.0px 0.0px 0.0px 0.0px; line-height: 16.0px; font: 14.0px Arial; color: #323333; -webkit-text-stroke: #323333}span.s1 {font-kerning: none

# Editor report: Acceptance letter

**DOI:** 10.1371/journal.pntd.0013770.r002

**Authors:** Angelica Arcanjo

Dear Dr Araujo,

We are delighted to inform you that your manuscript, "FC-Duplex IgG1 (HTLV-1/2): Qualifying the Flow Cytometry IgG1 Duplex Assay for differential diagnosis of HTLV-1 and HTLV-2 Infections," has been formally accepted for publication in PLOS Neglected Tropical Diseases.

We have now passed your article onto the PLOS Production Department who will complete the rest of the publication process. All authors will receive a confirmation email upon publication.

The corresponding author will soon be receiving a typeset proof for review, to ensure errors have not been introduced during production. Please review the PDF proof of your manuscript carefully, as this is the last chance to correct any scientific or type-setting errors. Please note that major changes, or those which affect the scientific understanding of the work, will likely cause delays to the publication date of your manuscript. Note: Proofs for Front Matter articles (Editorial, Viewpoint, Symposium, Review, etc...) are generated on a different schedule and may not be made available as quickly.

Soon after your final files are uploaded, the early version of your manuscript will be published online unless you opted out of this process. The date of the early version will be your article's publication date. The final article will be published to the same URL, and all versions of the paper will be accessible to readers.

For Research Articles, you will receive an invoice from PLOS for your publication fee after your manuscript has reached the completed accept phase. If you receive an email requesting payment before acceptance or for any other service, this may be a phishing scheme. Learn how to identify phishing emails and protect your accounts at https://explore.plos.org/phishing.

Thank you again for supporting open-access publishing; we are looking forward to publishing your work in PLOS Neglected Tropical Diseases.

Best regards,

Shaden Kamhawi

co-Editor-in-Chief

PLOS Neglected Tropical Diseases

Paul Brindley

co-Editor-in-Chief

PLOS Neglected Tropical Diseases
